# Pilot Study on the Reliability of the Coach's Eye: Identifying Talent Throughout a 4-Day Cadet Judo Camp

**DOI:** 10.3389/fspor.2020.596369

**Published:** 2020-12-07

**Authors:** Alexandra H. Roberts, Daniel Greenwood, Clare Humberstone, Annette J. Raynor

**Affiliations:** ^1^School of Medical and Health Sciences, Edith Cowan University, Joondalup, WA, Australia; ^2^Australian Institute of Sport, Canberra, ACT, Australia; ^3^Sport and Exercise Science, La Trobe University, Melbourne, VIC, Australia; ^4^Human Performance Centre, College of Health Sciences, University of Memphis, Memphis, TN, United States

**Keywords:** talent identification, coach, selection, inter-rater agreement, combat sport, potential, reliability – reproducibility of results, coaching (performance)

## Abstract

A typical assumption found in talent identification literature is that different coaches, given the same athletes and circumstances, will identify the same subset of athletes as “talented”. However, while coaches play a major role during talent identification in practical sport settings, there is limited empirical research exploring the processes which underpin this. The purpose of this study was to explore the reliability of “the coach's eye” during the assessment of talent in a group of athletes. Specifically, this project compared inter-coach agreement between nine judo coaches (ages 35.8 ± 10.6 years) with varying levels of experience (12.9 ± 8.9 years) in the evaluation of 24 talented cadet judo athletes (13–15 years) at seven timepoints throughout a 4-day development training camp. Without discussion of their scores with other coaches, coaches provided a single score representing each athlete's “potential for future performance” on an 11-point Likert scale at each timepoint. Scores from each coach were converted into rankings from 1 to 24 to create a normalized scale to facilitate comparison of athletes. Based on their rankings at each timepoint, athletes were placed into one of three evenly distributed groups (high, medium, and low rank). Inter-coach agreement at each timepoint was determined by the number of coaches who ranked each athlete in the same group, categorized at three levels: 50, 75 or 100% agreement. Overall results showed that at completion of the camp, coaches reached 100% agreement on only two athletes, both of whom were in the high rank group. When inter-coach agreement was set at 50%, 15 athletes (62.5%) were placed into like groups. The first timepoint at which coaches were able to differentiate between the majority of athletes was Timepoint 3 (end of day 2). The findings suggest that, in isolation, coaches do not agree on the talent or potential of athletes. This indicates that the “coach's eye” is subjective and variable, and, given the same context, there is poor inter-coach agreement in the identification of talented athletes. In turn, these findings may have significant implications for both future talent identification research and athlete selection processes by sport organizations.

## Introduction

Talent is rare, as only a small minority of people are talented (Baker and Wattie, 2018) and the forecasting aspect of talent identification makes the process of choosing who will succeed in the future challenging and relatively subjective (Johnston and Baker, 2020). Although historically, talent identification has been performed by coaches and/or scouts (Christensen, 2009; Bergkamp et al., 2019), in the last few decades there has been a shift toward creating evidence-based (i.e., empirically measurable) talent identification procedures in many sports. Interestingly, in measuring the effectiveness and accuracy of this empirical research, subjective coach decisions are often relied upon as the gold standard metric to which their results are compared (Roberts et al., 2019). This reliance on coaches within scientific investigations, along with the multifaceted and dynamic nature of talent (Vaeyens et al., 2008), indicates that coaches do, and will continue to play a significant role in the identification of sporting talent, both in the laboratory and on the field. In fact, the forecasting of future athlete performance is considered a major part of the coach's role, evaluating all aspects of an athlete (on and off the field) to forecast, or predict, who has the potential to be a high performer within a given sport (Tromp et al., 2013; Johansson and Fahlén, 2017; Roberts et al., 2020). However, the validity of coach decisions during talent identification is extremely difficult to determine due to the prognostic nature of these decisions, and the inherent de-selections that occur as part of the talent identification process. Without a formal assessment of coach accuracy and reliability, evaluating success or measuring talent identification methods over time will always be limited (Till and Baker, 2020). Despite anecdotal discussions, a fundamental unanswered question is; If viewed in the same context, would multiple coaches deem the same athlete(s) as “talented” and “untalented” when assessing their future potential?

There is limited understanding of how coaches determine an athlete's talent. Recent research has found that experiential knowledge plays a significant role in coach decision-making in strength and conditioning coaching and periodization (Till et al., 2019), during competition and training (Collins and Collins, 2016; Almeida et al., 2019), in return-to-play scenarios (Dawson et al., 2017) and talent identification (Roberts et al., 2020). If experience plays a role in these decisions, then it follows that coaches may identify and/or select athletes differently based on their own experiences. In many talent identification settings, decisions are made in short periods of time (days or hours) and are made by coaches with varying levels of expertise and experience, particularly at lower levels of performance and/or competition.

The inter-rater reliability, or agreement, of coaches has lacked attention in empirical research. The inherent assumption made when relying on coaches to perform talent identification is that a group of (relatively homogenous) coaches, when selecting from the same group of athletes under the same circumstances, will mostly agree on the evaluation of talent. That is, although “the coach's eye” (i.e., what coaches “see” when identifying talent) is understood to be subjective (Jokuschies et al., 2017), it will still result in similar decisions regarding the identification of talent. With this understanding, it is not expected that coaches would rank the athletes exactly the same, however, it is expected that coaches will reach a satisfactory level of agreement on the placement of athletes into like groups (for example, those with “low,” “medium” or “high” levels of potential). This study aimed to determine the level of agreement (i.e., inter-coach reliability) between a group of coaches throughout the course of a Cadet (youth) judo camp. Practically, given the same athletes, under the same circumstances, do coaches at a similar level (i.e., junior pathway coaches) come to the same assessment of athlete talent after 4 days? Specifically, did coaches agree on athlete placement within one of three ranked groups (high, medium, low) by the end of the camp.

## Methods

### Participants

Nine Australian judo coaches, four females and five males, of varying age (*M* = 35.8 ± 10.6 years); and experience (*M* = 12.9 ± 8.9 years) participated in this study (see Table 1 for full coach demographic information). All coaches have been identified by the National Sporting Organization as being skilled, up-and-coming coaches. Seven coaches were of Australian background, with one coach Japanese and one Brazilian. Ethical approval was obtained from the relevant Human Research Ethics Committees. All coaches gave their consent to be involved in the study and as no athlete data was collected, the Ethics Committees waived the need for informed consent from the athletes.

**Table 1:** Coach demographic information, including playing, coaching and education levels.

**Coach**	**Gender**	**Age**	**Judo coaching**	**Judo playing**	**Primary**	**Judo coaching**	**Other relevant education**
**identifier**		**(years)**	**experience (years)**	**experience (years)**	**coaching level**	**accreditation**	
A	F	22	3	10	Cadets	JA Coach Judo (Level 2)	B Health Science (Fitness)
B	M	43	20	10	Seniors	None	
C	M	53	30	20	Seniors	JA Senior Coach (Level 3)	
D	F	45	5	17	Cadets	IJF Academy Instructor Certificate (Level 1)	
E	F	24	9	7	Cadets	JA Coach Judo (Level 2); IJF Academy Coach Certificate (Level 2)	B Physiotherapy
F	F	21	1	4	Cadets	JA Assistant Coach (Level 1)	
G	M	41	15	25	Cadets	IJF Academy Coach Certificate (Level 2)	
H	M	35	13	18	Juniors	JA Coach Judo (Level 2)	B Physical Education
I	M	38	20	25	Seniors	JA Senior Coach (Level 3)	B Sports Science
**Totals**	5 F;5 M	35.8 ± 10.6	12.9 ± 8.9	15.1 ± 7.3	5 Cadets; 1 Juniors;
**(Range)**		(21–53)	(1–30)	(4–25)	3 Seniors

*JA, Judo Australia; IJF, International Judo Federation*.

### Procedure

This study was conducted at a 4-day “Judo Futures” development camp run by Judo Australia, consisting of skill/drill training, randori (sparring), recovery sessions, educational lectures, team-building activities and group meals. Fifty athletes were invited to be part of the camp based on their performance at the most recent National Championships. Twenty-five of the athletes at the camp were randomly selected for inclusion in the study as following pilot testing and coach comments about the practical process of identifying talent in unfamiliar athletes, this was deemed the maximum they could “evaluate” during the camp. Coaches were also assigned a mixture of coaching duties throughout the camp. Athletes were randomly assigned a number between 1 and 50, and the athletes that were evaluated in this study were those numbered between 1 and 25. For identification throughout the camp numbers were visible on their hands, feet and gi (jacket). The athlete designated as #3 did not attend the camp at the last minute, so the final number of athletes evaluated was 24. This included 13 male and 11 female judo cadets with a mean age of 13.88 ± 0.69 years. Athletes were unaware that the research was occurring to prevent potential observation-related behavior changes.

Coaches were asked to rate each athlete on an 11-point Likert scale, with 1 being “limited potential – unlikely to have a competitive future in sport”; 6 being “average potential – no more or less likely than peers to have a competitive future” and 11 being “extremely high potential – good potential to be a future Olympic medalist.” An 11-point scale was chosen to increase the generalizability and sensitivity of results when compared to smaller scales and is considered more appropriate for statistical analyses (Chyung et al., 2017; Wu and Leung, 2017). Having an odd-numbered scale (i.e., the inclusion of a mid-point) allowed coaches to express neutral opinions. All ratings were collected electronically using Qualtrics (Version January 2019, Qualtrics, Provo, Utah, USA).

The 4-day duration of the camp provided seven measurement opportunities. The first rating was made the conclusion of Day 1 (8 p.m.), with subsequent measures made in the morning prior to commencement of the first session of each day (~6–8 a.m.) and following the last session of each day (~8–10 p.m.). At each timepoint coaches received both an email and a text message with a link to the relevant survey which presented each athlete with their name, ID number and a photograph to facilitate recall. The athletes were presented one-by-one in a randomized order to remove any potential order biases. At each timepoint, coaches were instructed to “rate the athlete based on your *current overall* opinion of the athlete.” They were deliberately instructed to not base their score off the most recent session, but of their overall impression of the athlete up to that point. Coaches were provided the option of selecting “N/A” to indicate that they did not yet have enough information to rate a given athlete. This option was provided in order to avoid “misuse” of the midpoint as a “dumping ground” for those athletes they had not yet formed an opinion of, as recommended by Chyung et al. (2017). Importantly, coaches were blinded to their previous ratings for each athlete, and to the ratings provided by other coaches. Coaches were asked to provide their ratings independently of other coaches at each timepoint and to avoid discussions of scoring and athlete comparisons throughout the camp.

### Data Analysis

Data were analyzed descriptively using SPSS V26 (IBM Corporation, 2017). As coaches were given the option to use N/A if they did not have enough information to provide a rating for that athlete at that timepoint, coaches were included for statistical analysis at each timepoint if they rated 12 (50%) or more of the athletes. To standardize scores across coaches, the Likert score for each athlete was used to rank the athletes from 1 to 24 for each coach at each timepoint. If a coach rated two athletes with the same Likert score, they were given the same ranking.

Coaches were asked to score each athlete, rather than rank them or place them into the three groups, so as to avoid creating an artificial divide between groupings of athlete “potential.” By having coaches score the athletes over time, the organic emergence of separate groups allowed the research team to capture the time it took for coaches to discriminate between athletes of different potential levels.

For group analysis, rankings were used to sort athletes into three categories to conceptualize the talent identification process (Roberts et al., 2020). Therefore, the athlete rankings are presented in three groups – High ranking (athletes ranked from 1 to 8), medium ranking (9–16) and low ranking (17–24). The level of inter-coach agreement was determined by the percentage of coaches who placed the same athletes into the same groups, with three confidence levels used - 50% agreement (in which five or more coaches agreed on athlete placements), 75% agreement (seven or more coaches) and 100% agreement (all nine coaches).

## Results

This study sought to determine the level of inter-coach agreement between 9 coaches throughout the course of a 4-day judo camp. A total of 1,252 athlete ratings (out of a possible 1,512) were obtained over the 4-day camp (83%). The absence of ratings at several timepoints were due to selection of the “N/A” option, rather than a lack of response. Table 2 presents, for each timepoint, the number of coaches who rated more than 50% of the athletes (and were thus included in analysis for that timepoint). At no timepoint were all athletes able to be rated by each coach and for many coaches it took until the third session until they were able to rate the majority of the athletes.

**Table 2:** Number of coaches who rated more than 12 athletes (50%) for each timepoint.

**Timepoint**	**T1**	**T2**	**T3**	**T4**	**T5**	**T6**	**T7**
# Coaches	5	4	9	8	9	9	9

### Inter-Coach Reliability

**Figure 1:**
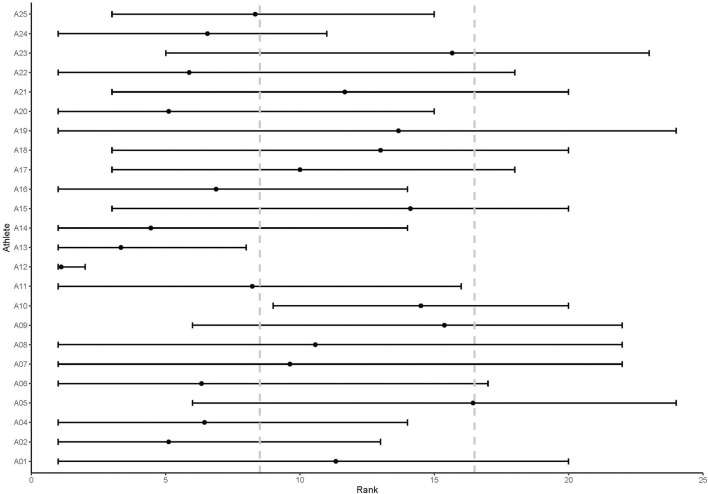
Range of athlete rankings by coaches at Timepoint 7. The range of each athlete's score is represented by the black horizontal line; dot on each horizontal line represents the mean rank for each athlete at Timepoint 7; gray vertical dotted lines depict the cutoff ranks for high-ranked (1–8), medium-ranked (9–16) and low-ranked (17–24) groups.

At T7 (end of day 4), there was a large range in the rankings for each athlete as presented in Figure 1. Coaches were able to reach 50% or higher agreement in the placement of athletes within their respective group (high, medium, low ranking) for 15 athletes (62.5%), 75% agreement on 5 athletes (20.8%), and 100% agreement on only 2 athletes (8.4%). It can be seen that even for athletes where the minimum agreement of 50% was reached there was considerable range in the rankings across coaches. For example, Athlete 6's rankings ranged from 1 to 13 and was therefore placed in the “high” rank group by six coaches (66% agreement), with the remaining three coaches ranking them in the “medium” (two coaches) and “low” (one coach) rank groups.

**Figure 2:**
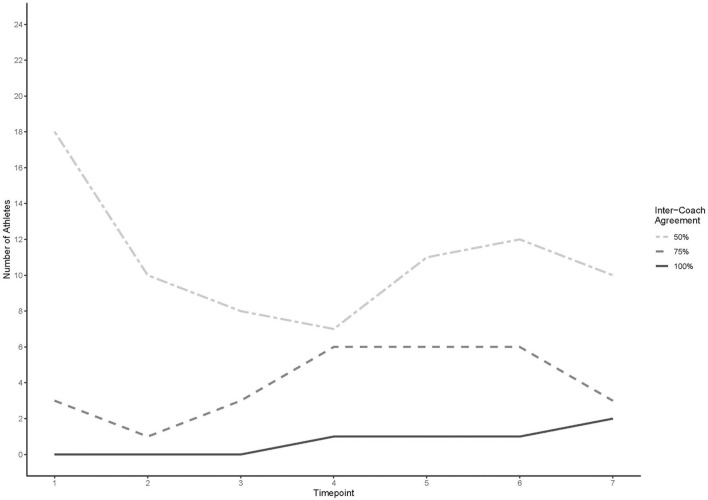
Number of athletes placed into like groups by coaches at three levels of inter-coach agreement.

Figure 2 presents the number of athletes included at each of the three levels of inter-coach agreement (50, 75, 100%) over the seven timepoints for all athletes rated at that timepoint.

### Sensitivity of Coach Judgments

**Figure 3:**
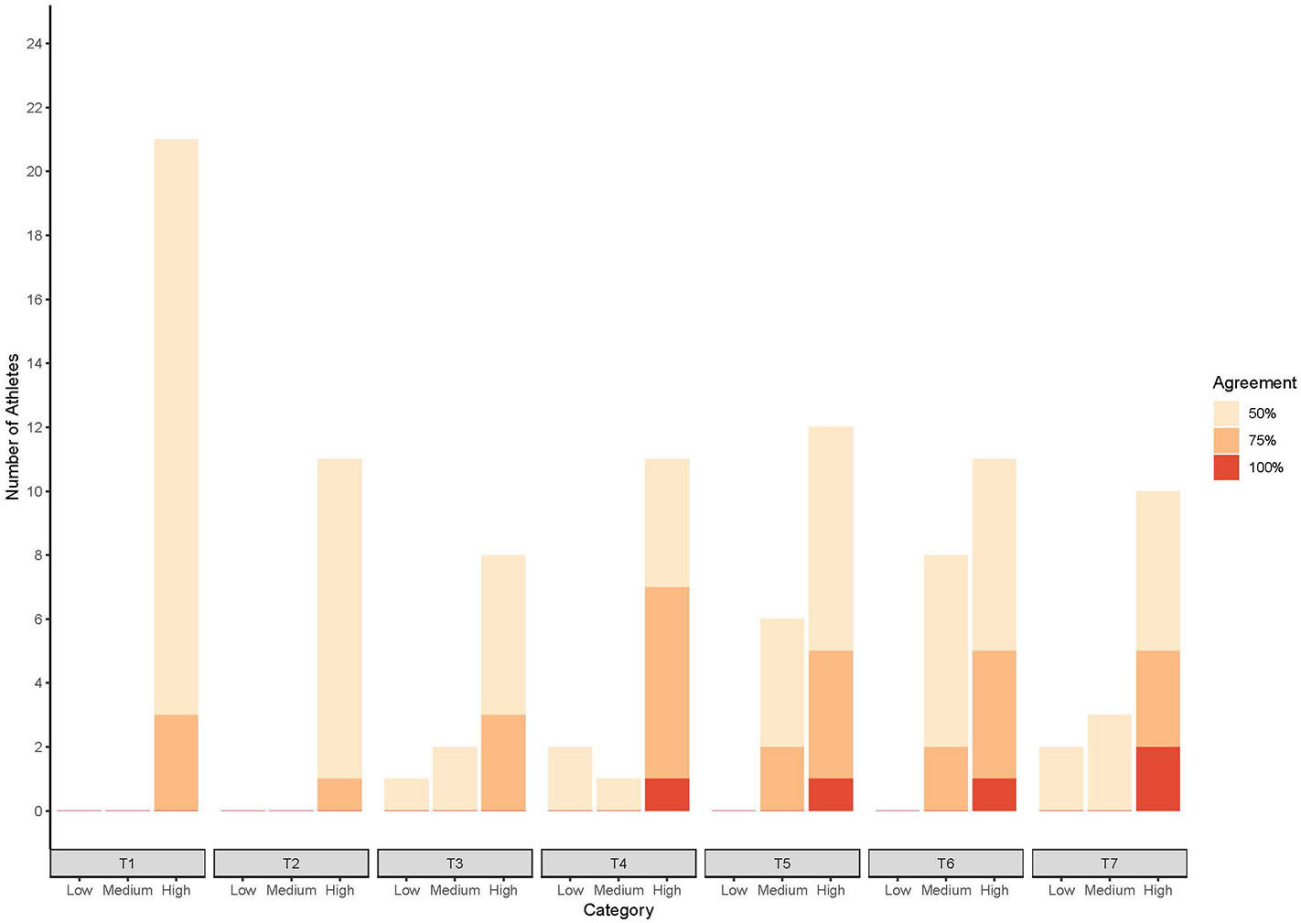
Number of athletes classified by group for each level of agreement across the seven timepoints.

When the inter-coach agreement was analyzed according to the three groups of athletes (high, medium, low rank), it was not until T3 that the coaches agreed on placing athletes in all three groups (Figure 3). T4 was the first time there was 100% inter-coach agreement for a single athlete and by T7, all coaches agreed on the placement of two athletes.

## Discussion and Conclusions

The purpose of this investigation was to test the assumption that different coaches within the same sport, given the same athletes and circumstances, will identify the same subset of athletes as being talented. Inter-coach reliability was measured through the level of agreement between a group of coaches in their assessment of athlete talent throughout and at the completion of a 4-day talent camp. Our key finding was that, given the same athletes, context and time, coaches did not assess talent in the same way. Specifically, a maximum of two athletes were placed into the same ranked group by 100% of the coaches at only one point throughout the seven measured timepoints.

The current findings emphasize the inconsistencies and a lack of reliability between coaches when identifying talent in cadet judo athletes. These results demonstrate that identifying talent is not as straightforward and reliable between talent arbiters as has been previously assumed. Using what could be considered a relatively low level of sensitivity (50% agreement) and placing athletes into one of three groups, coaches were unable to agree on athlete rankings, implying an absence of consensus among coaches as to which athletes should or should not be identified as talented.

Despite the homogeneity of athletes which were involved in the study, or perhaps because of it, it is apparent that coaches disagree about what a “talented” athlete looks like. This is in alignment with the findings of both Wiseman et al. (2014) and Tromp et al. (2013), in which experienced ice hockey coaches and scouts could not agree on rankings of athletes after a single tryout and eight games, respectively. Wiseman and colleagues state that traits such as “coachability” and “character” are important considerations during athlete selection that are unable to be observed in short, one-shot tryouts. From the current research, data showed that it was not until the end of the second day (T3) that coaches were able to distinguish between those with comparatively more or less potential. Prior to this, is appears that coaches were not able to sufficiently differentiate between athletes enough to sort them into three different groups. The current results highlight that even with extended exposure to athletes, in this case 4 days, the level of agreement between coaches on the evaluation of athlete's future potential was not necessarily “better” than in a one-off observation. It appears that it is easier for coaches to agree on the placemement of athletes in the “high rank” group, suggesting that athletes with more potential are more easily differentiated from their peers with less potential.

Interestingly, coaches either chose not to, or were not able to, rate every athlete at every timepoint, with coaches rating as few as four athletes at some timepoints, however for those included in the analysis they rated between 13 and 23 athletes per session. At no timepoint was every coach able to rate every athlete suggesting a “bandwidth” limit to the observational capacity of a coach during the identification process. This indicates that having a single coach evaluate multiple athletes is not an ideal scenario, as coaches are unable to form a consistent opinion of an athlete's talent that they are comfortable with. This finding supports previous research by Roberts et al. (2020), who found that coaches require time to get to know athletes before forming a confident evaluation of the athlete's future sporting potential. In this study, although the coaches had 4 days in which to evaluate the athletes, it appears that the number of athletes inhibited the coaches' capacity to survey the athletes in as much detail as they would require to make confident judgments and decisions.

This study captured the level of agreement between coaches with limited external influences, such as discussion with other coaches, providing a “natural” setting which, according to Stewart et al. (1997) is ideal for the study of judgment and decision-making. This is an important element of the current research, highlighting that without a discussion process consensus among coaches is all but impossible. This highlights why coaches may disagree with others' evaluations when selecting teams and why this can be such a point of friction within sporting organizations. The challenge of having multiple coaches decide on selection processes is clear from this research, demonstrating why many head coaches like to have the “final say” when identifying or selecting athletes. An important question for future research is to examine why is there such a difference in athlete evaluations between this group, and why in the past have we assumed that coaches will identify the same athletes under the same circumstances.

Coaches were not provided with any guidance as to what “talent” is defined as, nor what attributes may contribute. As such, the ratings provided their interpretation and application of the Likert scale, and subsequent rankings are entirely subjective based on the coaches' own knowledge and experience. While guidance could have been provided, previous research highlights that providing coaches with checklists or similar items does not necessarily increase inter-rater agreement (Wiseman et al., 2014), as judgments of performance are believed to be intrinsically subjective (Jako and Murphy, 1990). As such in the current study, coaches were instructed to score athletes based on their own perceptions of talent, rather than on specific guidelines or alignment with a given coach, thus providing a more ecologically valid identification scenario.

The AM and PM ratings provided evaluation of athletes on 12-h cycles. Interestingly, results show that different ratings were evident for the same athletes during the “overnight” cycles, when coaches had no interactions with the athletes. While one could argue that with no additional information gained overnight their assessment of each athlete should not have changed, these results highlight that there may be a reflective practice that the coach goes through, or a reduction in the effect of most recent interactions that occurred immediately prior to their evening evaluation, thus changing their assessment of talent. While outside of the scope of this paper, this finding aligns with the judgment and decision-making literature in that emotions (including tiredness) can affect evaluative judgments, as can age and gender (Weber and Johnson, 2009). These considerations will be important future steps in coach reliability research and in the practical talent identification processes adopted by sport organizations. The amount and experience of coaches present at these camps is always a limitation in this type of research, but the difference in perceptions between expert and novice coaches would also be an important research question. Investigation into the agreement of coaches of varying experience levels would be a valuable continuation of this research. Finally, an important question for future research is to examine why there is such a difference in athlete evaluations between coaches, and why in the past it has been assumed that coaches will identify the same athletes under the same circumstances.

A limitation of this study was that the camp used for data collection was not designed as a “true” talent identification or selection camp, as there was no official selection made following the camp. Rather, it was a development camp with pre-selected athletes and the coaches' primary responsibilities during the camp were to actively coach and develop the athletes, rather than to observe and evaluate. Their coaching responsibilities varied each day, and may have contributed to the low level of agreement at each timepoint and their inability to rate each athlete at each timepoint – on days when coaches were responsible for running sessions, they may not have been able to observe as many athletes as they otherwise would have. Coaches may also not have dedicated the same level of analytical thinking to these ratings as they would have if it had been a “real” identification scenario with athlete selection outcomes.

In conclusion, the process of talent identification, and the subsequent athlete selections, is undoubtedly complex. This research has added to the area by demonstrating that different coaches, when given the same athletes and circumstances, do not identify the same subset of athletes as being talented. Continuing to enhance our understanding of how coaches make talent decisions will help both coaches and practitioners become more aware of the necessary time and information needed for coaches to make confident, reliable decisions. This finding that coaches do not agree on the future potential of these athletes indicates that talent identification is not strictly related to athlete qualities which can be objectively measured. Instead, it is heavily dependent upon coach factors – possibly the “coaches eye” or “gut instinct” that are often spoken about in coaching literature. Exactly what these factors are is a question that remains for future research.

## Data Availability Statement

The raw data supporting the conclusions of this article will be made available by the authors, without undue reservation.

## Ethics Statement

The studies involving human participants were reviewed and approved by Human Research Ethics Committee, Edith Cowan University. The participants provided their written informed consent to participate in this study.

## Author Contributions

ARo, DG, CH, and ARa contributed to the design of the work, interpretation of the data, and revised the work critically. ARo acquired the data and drafted the work. All authors approved the final version to be published and agreed to be accountable for all aspects of the work.

## Conflict of Interest

The authors declare that the research was conducted in the absence of any commercial or financial relationships that could be construed as a potential conflict of interest.
